# Embodying Bounded Rationality: From Embodied Bounded Rationality to Embodied Rationality

**DOI:** 10.3389/fpsyg.2021.710607

**Published:** 2021-09-09

**Authors:** Enrico Petracca

**Affiliations:** School of Economics, Management and Statistics, University of Bologna, Bologna, Italy

**Keywords:** bounded rationality, moderate and radical embodied cognition, embodied rationality, embodied heuristics, Herbert Simon

## Abstract

Views of embodied cognition vary in degree of radicalism. The goal of this article is to explore how the range of moderate and radical views of embodied cognition can inform new approaches to rationality. In this exploration, Herbert Simon's bounded rationality is taken for its complete disembodiedness as a reference base against which to measure the increasing embodied content of new approaches to rationality. We use the label “embodied bounded rationality” to explore how moderate embodiment can reform Simon's bounded rationality while, on the opposite side of the embodied spectrum, the label “embodied rationality” is employed to explore how radical embodiment can more deeply transform the idea of what is rational. In between the two poles, the labels “body rationality” and “extended rationality” are introduced to explore how also intermediate embodiment can fruitfully inform the research on rationality.

## Distance From Simon'S Bounded Rationality As a Metric For the Embodiment of Rationality

In recent years, an increasing number of works have suggested that the study of rationality can be fruitfully informed by the idea of embodied cognition in cognitive science (e.g., Spellman and Schnall, 2009; Mastrogiorgio and Petracca, 2016; Gallagher, 2018; Viale, 2019; Gallese et al., 2020). Despite the mounting interest, no agreement seems to exist, however, about the intellectual foundations of such attempts at integration—the giants upon whose shoulders an embodied notion of rationality is supposed to stand. Which extant notion of rationality, if any, is taken as a reference “to embody?” How does this relate to the strand of embodied cognition selected for the task? These questions still await systematic investigation.

The research program called “embodied bounded rationality” (Gallese et al., 2020) has chosen to stand on the strong shoulders of Herbert Simon's bounded rationality from the very name. This choice has compelling reasons worthy of being mentioned. Introduced more than 70 years ago as the first cognitive science-based approach to rationality (Simon, 1947), bounded rationality has ever since represented a vehicle for introducing cognitive science advances into the study of rationality on a rolling basis. Indeed, scholars have continued to use Simon's label over the decades for bridging the gap with cognitive science, even if ending up proposing versions of bounded rationality quite different from Simon's original one (e.g., Kahneman, 2003; see Fiori, 2011).

On the basis of the above, seemingly no better giant than Simon could have been chosen for the task of embodying rationality. Nevertheless—it needs to be recognized—supporters of embodied cognition might have something to object to. As a founding father of what is called “cognitivism” (Haugeland, 1978), Simon conceived of cognition as a fundamentally abstract and disembodied phenomenon and was as such rather skeptical of embodied cognition since its inception. His skepticism rose to the point of publicly engaging in a controversy with early proponents of embodied cognition (Vera and Simon, 1993) in which his last, peremptory words were: “there is no need […] for cognitive psychology to adopt a whole new language and research agenda” (p. 46). Simon's role and significance in the history of cognitive science are crucial for our discourse, as the entire project of embodied cognition set in motion as a reaction to his cognitivism (Agre, 1993; Petracca, 2017), and the aim to go beyond cognitivist assumptions possibly remains today the only common trait of the many and diverse approaches within embodied cognition.

How, then, to reasonably recruit Simon and his bounded rationality for a project pursuing the embodiment of rationality? Doing so would require, we argue, rethinking Simon's role from that of the godfather—an unfit role for the reasons above—to that, less symbolic but more operational, of a “conceptual yardstick.” What does it mean? Because of its fundamental disembodiedness, we suggest taking Simon's bounded rationality as the level zero of a virtual embodiment scale for rationality, which can then be used to assess whether and to what extent new and extant notions of rationality exhibit embodied content. In a nutshell, we suggest using the conceptual distance from Simon's bounded rationality as a metric of embodiment in the field of rationality.

Taking the distance from Simon as a measure of the embodiment of rationality is not conceptually different from what scholars of embodied cognition already do to sort different positions within their own field. Indeed, it is today customary to categorize strands of embodied cognition according to their degree of radicalism (see Goldman and de Vignemont, 2009; Gallagher, 2011), which is just another way to sort them according to their distance from cognitivism. This kind of reconstruction traditionally individuates two poles in a spectrum of positions: a “moderate” embodied cognition that aims to reform cognitivism through selective embodied add-ons and a “radical” embodied cognition that rejects cognitivism as providing no benchmark whatsoever for cognitive activity^1^. In between, a variety of positions target one or more aspects of cognitivism with the aim of either reforming or rejecting them.

The goal of this article is to explore how the range of varying-in-attitude embodied positions may inform new views of rationality. To do so, we first suggest rationalizing the use of two labels currently employed interchangeably in the literature, “embodied bounded rationality” (Viale, 2019; Gallese et al., 2020) and “embodied rationality” (Spellman and Schnall, 2009; Mastrogiorgio and Petracca, 2015, 2016; Gallagher, 2018), by tying each to a different degree of embodied radicalism. By its very name, embodied bounded rationality seems close to Simon's original notion and for this reason especially suited for pursuing a reformistic (embodied) approach. On the opposite side of the spectrum, embodied rationality may be a vehicle for radical (embodied) positions that altogether reject the central tenets of cognitivism—notably, mental representationalism and computationalism—and do not intend to use them for the study of rationality. In between these poles, we also identify two possible intermediate approaches. The one, called “body rationality,” is intended for studying the body foundations of cognitive and reasoning shortcuts such as heuristics; the other, “extended rationality,” is instead aimed to integrate into rationality insights from the research on extended cognition. To be clear on the increasing order of radicalism, the range goes from embodied bounded rationality through body rationality and extended rationality, and finally gets to embodied rationality. As we will show, the more radical the view of embodied cognition we adopt, the more deeply we will need to rethink the current definition of rationality.

As for what we mean by current definition of rationality, a clarification is required. Although different ideas of what is rational are lumped together under the bounded rationality banner, there is a common core to most of them: the idea that agents' rationality fundamentally lies in their successful adaptation to task environments. Adaptation is the same normative principle underlying Simon's bounded rationality, Gerd Gigerenzer's ecological rationality, and Markus Raab's motor heuristics, although they may differ in the details of adaptation. In this article, adaptation is taken as the higher-order definition of rationality that we will attempt to embody and, at radical embodied latitudes, possibly overcome. In the process, we will also discuss non-adaptive views that understand rationality more traditionally as logical or probabilistic inference, such as Daniel Kahneman's, but broadly construed adaptationism and its possible embodiment(s) will be our primary concern.

The article proceeds by introducing the four notions of rationality in increasing order of embodied radicalism or, equivalently stated, in increasing distance from Simon's bounded rationality. Section Embodied Bounded Rationality: The Reformist Embodied Approach to Bounded Rationality introduces embodied bounded rationality. Sections Body Rationality: The Bodily Roots of Adaptive Heuristics and Extended Rationality: Extended Cognition and Un-Bounded Rationality are devoted respectively to body rationality and extended rationality. Then, section Embodied Rationality: The Radical Embodied Approach to Rationality discusses embodied rationality. Section Discussion and Conclusion concludes by providing some comparative remarks.

Before moving on to the discussion, doing some justice to Simon is in order. Although Simon's thought is presented here as the quintessence of disembodiedness, we will also see that over his long career he foreshadowed, although only sketchily, some of the topics that would later be addressed by students of embodied cognition. A further reason why Simon represents, we argue, an inescapable reference for any contemporary discussion of rationality.

## Embodied Bounded Rationality: The Reformist Embodied Approach To Bounded Rationality

Alvin Goldman has introduced the term “moderate embodied cognition” (Goldman, 2012) as an umbrella for those views of embodied cognition variously compatible with cognitivism. As Goldman claims, his position is moderate in so far as “while highlighting the pervasiveness in cognition of bodily factors, it does not invoke this as a ground for revolutionizing the methodology of cognitive science” (Goldman, 2012, p. 71). Such non-revolutionary intent dovetails quite perfectly with Simon's above-mentioned plea not to change the language and research agenda of cognitivism under pressure from embodied cognition (Vera and Simon, 1993). If moderate embodied cognition has provided a convenient banner for moderate, reformist embodied steps beyond cognitivism, the banner “embodied bounded rationality” (Gallese et al., 2020) may prove to be convenient as well, we argue, for moderate, reformist embodied steps beyond Simon's bounded rationality. This section is devoted to sketching what the reformism of embodied bounded rationality consists of.

### From Abstract to Embodied Representations

Much of the debate about cognitivism revolves around the subject of mental representations and variously concerns their existence, nature, role, extent, manipulation, sufficiency, and/or necessity (see Pitt, 2020). While retaining mental representations as a requirement for cognition, the moderate embodied approach is deemed to be a “genuine rival” (Goldman and de Vignemont, 2009, p. 154) of cognitivism in so far as it challenges the disembodied nature of representations. It rejects, in particular, the existence of an abstract, amodal (machine) code of the mind which Fodor (1975) famously called “language of thought,” and posits instead that mental representations are rooted in sensorial, motoric, interoceptive (e.g., visceral), and affective neural resources (e.g., Gallese, 2005; Barsalou, 2008; Meteyard et al., 2012) called, in short, “B-formats” (Goldman and de Vignemont, 2009). Currently, a debate exists between those—who may be called the “moderate moderates”—who think of B-formats as just one type of representations alongside amodal ones (e.g., Goldman and de Vignemont, 2009) and those—the “not-so-moderates”—who suggest that all representations are embodied one way or another (e.g., Gallese and Lakoff, 2005). Given this background, moderate embodied cognition can inform embodied bounded rationality suggesting the latter to set its main goal in reforming the amodal representationalism of bounded rationality without putting representations themselves into question.

Before taking this path, some preliminary grasp on the nature of representations in Simon's bounded rationality is needed. What we may expect of any representational approach to rationality is a framework in which agents manipulate their mental representations in some way considered “rational.” As Fodor defined it, capturing the gist of what just said, rationality is the “organism's intelligent management of its representational resources” (Fodor, 1975, p. 169). On this view, the core of rationality lies in *how* organisms manage their representations, that is, how well they do so when assessed against a given normative principle. There is no doubt that what just said fits well Simon's idea of rationality (see Simon, 1955, in which the normative principle is represented by an agent's aspiration level), but probably this is not all there is in his thought. Simon not only understood representations as the contents of cognitive activity but also as means for meta-representing cognitive activity. In other words, representations play in Simon also a meta-representational role in so far as they allow the *simulation* of agents' cognitive activity. What Simon calls “symbols”—abstract patterns that obey the rules of formal systems (also called “physical symbol systems”)—are pluripotent vehicles able to produce second-order representations, that is, representations of agents' representations (Newell and Simon, 1972). Integrating this meta-representational approach into the study of rationality, Simon and his colleague Allen Newell enunciated the so-called “physical symbol system hypothesis,” according to which “a physical symbol system has the necessary and sufficient means for general intelligent action” (Newell and Simon, 1976, p. 116). In other words, Newell and Simon consider symbols necessary and sufficient conditions for any form of rational manipulation of representations and simulation thereof. Disentangling the representational from the meta-representational side of Simon's approach is crucial, we argue, to settle a persistent interpretative controversy over his thought. In the controversy, Felin et al. (2017) consider Simon assuming agents' perceptual omniscience, that is, their capacity to build potentially perfect representations of their environments. Instead, Gerd Gigerenzer and colleagues contend that Simon held a species-specific—far from omniscient—idea of perception (Chater et al., 2018, p. 803–806). To reconcile these views, one likely needs to acknowledge that in Simon's framework what is omniscient and perfect are meta-representations, not representations themselves. Omniscient meta-representations can simulate an endless variety of phenomena at the lower level of agents' representations, from species-specific cognition to any form of perceptual and reasoning bias.

This closer look at Simon is useful if we want to discuss embodied representations. On the one hand, meta-representations seem even harder to reconcile with embodiment as they are of a higher order of abstractness than mental representations. On the other hand, however, Simon's “simulationism” evokes suggestive linguistic proximity to moderate embodiment since the neural mechanisms thought to be at the root of B-formats are called “embodied simulations” (Barsalou, 2008; Gallese and Sinigaglia, 2011; Goldman, 2012). But before expecting too much of this linguistic glimmer, it is important to remark that the two ideas of simulation are quite different. While in Simon a simulation is a method to model cognitive activity, an embodied simulation is instead defined as the “[neural] reenactment of perceptual, motor, and introspective states acquired during experience with the world, body, and mind” (Barsalou, 2008, p. 618). In other words, what in Simon is a methodological approach is instead a specific neural mechanism in moderate embodied cognition.

If embodied bounded rationality aims to follow the footsteps of moderate embodiment, it needs to leave meta-representations behind and go down the neural path. In this regard, Barsalou (2008) distinguishes between two main neural types of embodied simulation: a “cognitive simulation” and a “social simulation.” In cognitive simulation, B-formats are used, among other things, to ground and structure concepts (see Harnad, 1990). For instance, the originally purely sensorial notion of “coldness” is neurally reenacted (in sensory-motor areas) to acquire the affective meaning of “emotional coldness” (e.g., Lakoff and Johnson, 1999). In social simulation instead, B-formats ground and enable social faculties such as mind-reading (this is thought to happen mostly *via* mirror mechanisms). Importantly for our discussion, theories of cognitive simulation seem to be more focused on the representational role of B-formats than theories of social simulation, which are instead more interested in B-formats' function (Gallese and Sinigaglia, 2011, p. 517). For this reason, cognitive theories appear to be more immediately relevant if the aim is to go beyond amodal representationalism. In this regard, Barsalou (1999) has introduced the framework of “perceptual symbol systems” as a way to comprehensively ground Simon's physical symbol systems into embodied simulation. Rather than being amodal, representations are on Barsalou's account entirely rooted in sensorial, motoric, interoceptive, and affective neural systems. In what follows, we will see how embodied moderatism concerns not only the nature of representations but also the very definition of rationality.

### Embodied Moderatism and the Definition of Bounded Rationality

So far, the moderatism of embodied bounded rationality has consisted in retaining mental representations by reforming their nature. This section suggests that to unleash the full potential of embodied representationalism, the discussion has to take on directly the definition of bounded rationality. Otherwise, we would find ourselves in the curious situation in which embodied bounded rationality is moderately embodied but is not really bounded rationality.

Exegetical quarrels aside, the gist of Simon's bounded rationality lies in the idea that rationality is the outcome of a process of adaptation of agents' bounded representations and computations to the demands of task environments (Simon, 1955, 1956). Simon conveyed this adaptive message through the famous metaphor of a pair of scissors:

Just as a scissors cannot cut paper without two blades, a theory of thinking and problem solving [i.e., a theory of rationality] cannot predict behavior unless it encompasses both an analysis of the structure of task environments and an analysis of the limits of rational adaptation to task requirements (Newell and Simon, 1972, p. 55).

Another way to put the metaphor is to say that the rationality of individuals does not depend on absolute cognitive resources but on the adequacy of those resources to task demands (Callebaut, 2007). Ants possess very limited cognitive resources if considered in absolute terms, but assessing them this way would prevent us from realizing that they are enough for ants to succeed—i.e., to survive—in their environment (Simon, 1996a). In Simon's view, organisms are hardwired with, but can also acquire developmentally, undemanding criteria and procedures for decision-making and problem-solving—called heuristics—that permit them to succeed in their environments. The simple but path-breaking idea that rationality lies in the use of adaptive heuristics rather than optimal procedures (Simon, 1955) continues to inspire the current studies on bounded rationality. The goal of a major contemporary strand of research, called ecological rationality, is to make a catalog of the “fast-and-frugal” heuristics used by individuals to make decisions and study in which environments they work (Gigerenzer et al., 1999).

It is crucial for our discourse to recognize that embodied simulations are resource-saving neural mechanisms the same way heuristics are resource-saving cognitive mechanisms. Evolutionarily, heuristics and embodied simulations are two sides of the same coin of adaptation. The parallel between heuristics and embodied simulations was explicitly drawn in Gallese and Goldman (1998)—the first article to hypothesize that the mirror mechanism is a more deeply-rooted neural mechanism for mind-reading than theory of mind. In fact, Gallese and Goldman call the embodied simulation occurring in the mirror system a “simulation heuristic.” As they put it,

MN [mirror neuron] activity seems to be nature's way of getting the observer into the same “mental shoes” as the target—exactly what the conjectured *simulation heuristic* aims to do. […] Our conjecture is only that MNs represent a primitive version, or possibly a precursor in phylogeny, of a *simulation heuristic* that might underlie mind-reading (p. 497–498, italics added).

The meaning of heuristic in this passage is virtually the same as Simon's: embodied simulation is considered to be a species-specific (although not restricted to humans), hardwired mechanism that allows individuals to perform the complex mental faculty of mind-reading fast and frugally. Fastness would be guaranteed by the automaticity of embodied simulation, and frugality by the reuse of sensory-motor resources. On the basis of this, an analogy between Gallese and Goldman's simulation heuristic and theory of mind on the one hand, and heuristics in general and optimal criteria for decision-making and problem-solving on the other hand, does not seem too far-fetched. To complete the analogy, as using heuristics does not preclude resorting to more demanding decision-making and problem-solving mechanisms when need be, the use of simulation heuristics does not likewise preclude resort to more demanding mind-reading mechanisms whenever useful. In both cases, it is situational factors that ultimately decide on the rationality (i.e., adaptivity) of the mechanism.

Other embodied simulation mechanisms can be compared to heuristics. Consider metaphorical simulation discussed above, in which originally sensory-motor, interoceptive, and affective resources are reenacted in wider target domains (Lakoff and Johnson, 1999). Metaphors do not merely structure concepts but, more exactly, do so in a way that saves neural and conceptual resources. In the metaphorical judgment “this person is cold” we can see in action a resource-saving mechanism that reuses a sensorial resource for affective purposes. As such, a metaphorical judgment can be considered a form of simulation heuristic the same way it is understood by Gallese and Goldman. This line of argument can also extend to decision-making, where metaphors like “heavy decision” or “balanced decision,” hinging on the sensory-motor notions of physical weight and physical balance, influence decision-making in the way of making it, again, fast and frugal (see Lee and Schwarz, 2014).

Bounded rationality, however, is not only adaptation. In the post-Simonian version of Daniel Kahneman and Amos Tversky, the normative benchmark of bounded rationality is not environmental fitness but rather the axioms of logic and probability. In this approach that focuses on only one blade of Simon's scissors—limited cognition—, individuals' ability to be rational, i.e., to satisfy the axioms, is seen constantly threatened by perceptual imperfections, cognitive biases, and the use of misleading heuristics (Kahneman, 2003; Fiori, 2011). As this is the currently prevailing interpretation of bounded rationality, the reformism of embodied bounded rationality should say something about it as well. Having no interest in meta-representations and hinging on perception, Kahneman and Tversky's view more naturally than Simon's can join forces with moderate approaches to perception. Barsalou (1999), for instance, discusses how encoding information in different perceptual modalities may give eventually rise to cognitive biases. In his account, embodied simulations are far from perfectly representational as, he says, “simulations are typically partial recreations of experience that can contain bias and error” (Barsalou, 2008, p. 620). Moreover, Kahneman and Tversky introduced their own “simulation heuristic” (Kahneman and Tversky, 1982), presented as a modified version of the more famous availability heuristic. Instead of merely using the ease of retrieving past events to infer their probability (as availability heuristic does), it is the ease of constructing mental representations and counterfactuals that simulation heuristic uses to infer probability. Gallese and Goldman (1998, p. 496) explicitly acknowledge that Kahneman and Tversky's simulation heuristic, particularly when used to construct representations of others' motives and actions, may be founded on their same inter-subjective notion of embodied simulation. Recently, Kahneman has established his view of bounded rationality upon dual-process theories of cognition (Kahneman, 2011), although this new foundation has hardly rendered the approach less disembodied. Petracca (2020) discusses how the slowness and fastness of judgments and decisions can be better understood in the context of embodied mechanisms that also involve embodied simulation.

## Body Rationality: The Bodily Roots of Adaptive Heuristics

Inherent to moderate embodiment is the “neurocentric idea that cognitive states are exclusively realized in neural hardware” (Alsmith and De Vignemont, 2012, p. 5). Such neuro-centrism—often understood as plain brain-centrism—may give rise to a concern about the triviality of embodiment. If cognition were considered to be embodied merely because the brain is part of the body, this would clearly render the embodiment claim trivial. Goldman and de Vignemont (2009) say that to avoid this risk many theorists have come to understand embodiment more specifically in terms of “the whole physical body minus the brain” (p. 154), or, as Damasio (1994) called it, in terms of the “body proper.” On their part, moderate theorists find likewise trivial the idea that cognition depends on features of the body, and although they admit that certain body states (such as postures) causally affect cognition, this is not deemed sufficient for considering the body proper a constitutive part of cognition (Goldman and de Vignemont, 2009)^2^. The approach that focuses on the role of the body proper for cognition, called biological embodiment (Gallagher, 2011; see Shapiro, 2004; Gibbs, 2005), represents a sort of intermediate position in the research on embodiment, halfway between neurocentric and more radical views that we will discuss in detail in section Embodied Rationality: The Radical Embodied Approach to Rationality^3^. This section is devoted to exploring how biological embodiment can inform a new approach to bounded rationality that we call “body rationality.”

In a sense, Gigerenzer and colleagues' ecological rationality (Gigerenzer et al., 1999) can be considered an as much intermediate position in the field of rationality. On the one hand, supporters of ecological rationality see themselves as heirs of Simon's tradition in its “purest form” (Gigerenzer et al., 1999, p. 14), as they subscribe to Simon's adaptive, scissors-like view of rationality (see also Gigerenzer and Goldstein, 1996). Moreover, they subscribe to Simon's computational program and follow “Simon and Newell's emphasis on creating precise computational models [of heuristics]” (Gigerenzer et al., 1999, p. 26). On the other hand, however, there is a point—an important one—on which ecological rationality does not seem to follow exactly in Simon's footsteps: mental representationalism. As it has been noticed, in ecological rationality

Mental representations […] are not abandoned, but the fact that simple processing solutions exploit structure in the environment does suggest the possibility of a weaker reliance on internal models of the world (Brighton and Todd, 2009, p. 341).

While the role of mental representations in ecological rationality is the object of debate (Petracca, 2017), as its proponents continue to use them for describing cognitive activity (e.g., Gigerenzer et al., 1991), it is otherwise uncontroversial that ecological rationality is on the whole less dependent on mental representationalism than Simon's bounded rationality. This point suggests that it may be a particularly good candidate for building a bridge with biological embodiment.

One simple remark can show why this is the case. There seems to be much more truth than meets the eye in heuristics being also called “rules of thumb.” In this expression, the thumb is understood as a resource of the body—a somatic device—that is used for measuring, making judgments, drawing inferences, making decisions, and solving problems (Mastrogiorgio and Petracca, 2015, 2016; Gallese et al., 2020). This remark suggests that it is possible to envisage an entire research program that studies the bodily roots of adaptive heuristics, that is, devoted to identifying those evolutionary and developmental processes that have led structural features of the human body—such as the thumb—to be used for adaptive purposes. The mildly representational view of heuristics in ecological rationality may provide a good starting point for such a new program. A program that can aspire even to reform Simon's scissors metaphor itself: if we put the body in the spotlight, the scissors of bounded rationality result no longer merely double-bladed (composed of cognition and environment) but also comprise a pivot, the body proper, which holds the blades together as an evolutionary and developmental interface (Mastrogiorgio and Petracca, 2015, 2016; Gallese et al., 2020).

In the embodied cognition literature, the body proper has mostly been understood in two ways: as a constraint and as a computational resource (Shapiro, 2019). However, these views are not mutually exclusive as inherent in the idea of a constraint is the complementary idea that it can become an opportunity in the right circumstances. Seen through the lens of the constraint/opportunity duality, it is easy to see how the thumb, along with other somatic devices, can be at the root of normative processes of rule-building. Consider, for instance, the role of somatic devices in the construction of measurement systems (Gibbs, 2005). While somatic devices are usually understood as body resources able to off-load the burden of individuals' cognition (Risko and Gilbert, 2016), they can also substitute for external resources. Over evolutionary and developmental timespans, thumbs and feet have in fact served as “on hand” embodied rulers for measuring or estimating features of the surrounding environment, eventually becoming standard units of measurement (i.e., an inch or a foot).

Proffitt and Linkenauger (2013) provide a productive framework for understanding the role of the body proper in cognition. What they call “phenotype” is deemed to include the three dimensions along which the body proper shapes cognition: the morphological, physiological, and behavioral dimensions. The way the body shapes measurement systems in the example above concerns prominently body morphology that, although being traditionally the least explored embodied dimension, is the one specifically investigated by Proffitt and Linkenauger. In particular, they study the role of body morphology in perception, and do so in two ways: in terms of morphological invariance (e.g., considering five-fingered hands as morphological invariants of the human species) or in terms of individual differences (e.g., considering hands' morphological variations between individuals). Interestingly for our argument, there is a distinct pragmatist undertone in Proffitt and Linkenauger's investigation as they emphasize how body morphology, along with the other phenotypical dimensions, modulates perception in ways that subserve agents' situational goals. For instance, in a task that involves grasping, they say that “apparent distances are scaled with morphology, and in particular, to the extent of an actor's reach or the size of his or her hand” (p. 172).

Gigerenzer's ecological rationality is naturally suited to be understood through the lens of biological embodiment as in some (rare) cases it is already biologically embodied. In the vast repertoire of fast-and-frugal heuristics, Gigerenzer (2007) discusses the “gaze heuristic,” which applies whenever individuals try to intercept an object, such as a ball, flying in the air. To trace mathematically the trajectory of the ball one should virtually compute differential equations, which is almost impossible to do (just literally) on the fly. To make the catching job done, the gaze heuristic provides alternative fast-and-frugal rules: “[f]ix your gaze on the ball, start running, and adjust your running speed so that the angle of gaze remains constant” (Gigerenzer, 2007, p. 7). No need to say that these rules are but rational reconstructions of what individuals unknowingly do every time they try to catch a flying ball. The gaze heuristic belongs to that class of fast-and-frugal heuristics that Markus Raab has recently called “motor heuristics” (Raab, 2017), which, concerning specifically the use of the body proper, are biologically embodied by definition.

There are, however, more subtle (but no less pervasive) forms of biological embodiment of fast-and-frugal heuristics. Consider, for instance, the “theory of prominence” (Albers, 2002) and the “QuickEst” heuristic (Hertwig et al., 1999), two judgment processes that exploit so-called “prominent numbers” (1, 2, 5, 10, 20, 50, 100, etc.) in the 10-based number system for fast-and-frugal numerical estimations. Here we are not interested in whether numerical prominence leads to reliable estimates or estimation biases, but in the origins of prominent numbers^4^. Again, fingers and hands feature prominently in this discussion. It is well known, but sometimes not sufficiently appreciated, that the 10-based number system originates in counting processes based on the 10 fingers of the hands (Gibbs, 2005). This leads to plausibly hypothesize that numerical accessibility and prominence have precise roots in body morphology (see also Lakoff and Núñez, 2000). As another instance, consider the 1/N heuristic (Gigerenzer and Gaissmaier, 2011), an evaluation and choice criterion that “weights” different options equally. The very ideas of weighting, pondering, and balancing when used in judgment and decision-making are, as seen, instances of embodied metaphors (Lee and Schwarz, 2014). Rarely, however, it is asked where the accessibility and cognitive relevance of the idea, say, of equal-weight comes from. A biologically embodied answer is that it originates in the morphological symmetry of the body, in the vestibular system, and in the sense of balance it controls (Gibbs, 2005). Similar considerations can be extended to entire classes of heuristics with the aim of uncovering their bodily, and particularly morphological, roots.

## Extended Rationality: Extended Cognition And Un-Bounded Rationality

Biological embodiment is not alone in populating the conceptual space between embodied moderatism and radicalism. The approach of extended cognition pioneered by philosopher Andy Clark also contends for that space. This raises the issue of relative positioning: which one is more leaning toward radicalism? As extended cognition posits that cognitive processes are neither bounded to the brain nor even to the body but also realize through resources of the environment—so going beyond biology as a requirement for cognition—this might suffice, we argue, to consider extended cognition more radical than biological embodiment. However, although it is sometimes presented as a radical position *per se* (e.g., Wilson and Clark, 2009), there are reasons to doubt that this is the case. Clark is well known not to reject mental representationalism and computationalism as he attempts to retain them—however limiting their extent (Clark and Toribio, 1994)—in an integrated framework known as “extended functionalism” (Clark, 2008; Kiverstein and Clark, 2009). According to this framework, what renders a resource cognitive is not its spatial location, inside or outside the body, but its function in the cognitive system (Clark and Chalmers, 1998; Clark, 2008). On this view, notebooks and hippocampal neurons can be seen as functionally equivalent to the extent they both support memory. This section explores how extended functionalism can inform bounded rationality and uses the banner “extended rationality” for the task.

Luckily, Clark has completed much of the preparatory work for us. Particularly in the early stages of extended functionalism, he discussed at length how his view might relate to Simon's. Unusually for a post-cognitivist scholar, he did not criticize Simon for his cognitivism but was even open to recognizing him as a forerunner of extended cognition. “Simon saw, very clearly,” Clark says, “that portions of the external world often functioned as a non-biological kind of memory. He thus saw a deep parity (parity, not identity) that can obtain between external and internal resources” (Clark, 2001, p. 139). Clark adds, however, that instead of extending the notion of self to include external resources, “Simon chose to go the other way” (Clark, 2001, p. 139), that is, he shrank the self so much that functions realized through external resources, like memory, ended up being out of its domain. When Clark goes on discussing Simon's bounded rationality, it is only coherent that he considers this concept “probably the first step” (Clark, 1998, p. 184) in the direction of recognizing the importance of external resources for rationality, yet an “insufficiently radical” (Clark, 1998, p. 243) step^5^.

According to Clark, there are two main routes for embodying rationality. One is what he calls “biological cognitive incrementalism,” a view “according to which full-scale human rationality is reached, rather directly, by some series of tweaks to basic biological modes of adaptive responses” (Clark, 2001, p. 122). As an instance of a basic biological adaptive response, one may think of the already mentioned intuitive use of the thumb for making spatial inferences, an intuitive method which can be eventually “tweaked” into becoming a formal heuristic (e.g., Wong, 2006). As such, biological cognitive incrementalism seems to be in full continuity with biological embodiment and body rationality, and it is not by chance that Clark discusses Gigerenzer's ecological rationality right in this context (Clark, 2001, p. 130). An alternative route—clearly Clark's favorite—for the embodiment of rationality goes instead down the path of extended functionalism. As human cognition is increasingly constituted—not just enabled—by external technological artifacts, the boundaries between biological and non-biological cognitive requirements become blurred. This acknowledgment, Clark suggests, should accordingly turn the discussion of rationality from biological to non-biological cognitive incrementalism.

In recent years, the research on extended cognition has shifted its focus from the study of functional “parity” (e.g., between notebooks and hippocampal neurons) to that of functional “complementary.” Functional complementarity means that external resources are not only employed as substitutes for internal resources but can also integrate with the latter in order to enhance individuals' overall cognitive capacity. The subtitle of Menary's (2007) book *Cognitive Integration: Mind and Cognition Unbounded* explicitly suggests that by using external resources cognition can become “unbounded.” Rehearsed in the domain of rationality, Menary's unboundedness seems to be rather in contrast—the opposite actually—to Simon's cognitive boundedness, and therefore induces one to wonder whether it is the case that cognitive complementarity leads in the end to an unbounded notion of rationality. As the idea of cognitive unboundedness seems suspiciously reminiscent of the omniscience of rational choice theory that Simon fought (with merit) throughout his career, it is of utmost importance to clarify this point in what follows.

The risk of mistaking the unboundedness of extended cognition as a restoration of rational choice theory occurs only if we adopt a non-adaptive framework. Consider Kahneman's non-adaptive bounded rationality, according to which humans would be fully rational if only they did not use misleading heuristics and were not ridden with cognitive biases. In Kahneman's framework, it is quite natural to think of external resources—understood as “cognitive artifacts” (see Hutchins, 1999)—as means to fix cognitive imperfections and get a step closer to the desired omniscience. But in Clark's framework omniscience does not play any role, not even as a benchmark (Clark, 1998). If it is true that coupled with external resources memory and other cognitive faculties can become virtually limitless (instead of a notebook, think of the far greater potential of a smartphone), the point Clark and other theorists of extended cognition would still raise is: is omniscience desirable from an ecological point of view? Or, is omniscence even meaningful once we come to understand what cognitive faculties are really for (see Glenberg, 1997)? This ecological tone, Arnau et al. (2014) have recently claimed, brings extended cognition quite close to ecological rationality: in both perspectives, it is rightly noticed, it is the environment and the task at hand that ultimately decide whether more cognitive capacity is beneficial or not. However, although ecological rationality and extended cognition undeniably share the ecological viewpoint, the way they deal with environments makes the two perspectives hard to integrate. The view of adaptation supported by theorists of extended cognition is far from the static and passive process envisioned by Simon and Gigerenzer. Clark's idea of adaptiveness is fundamentally active, so much that another name for his extended approach is “active externalism” (Clark and Chalmers, 1998). In Clark's view, individuals use *actively* the resources of environments to get a step closer to the kind of adaptive, circumstantial unboundedness envisioned by Menary (2007).

Importantly in the active kind of adaptation, individuals do not merely use environmental resources but altogether transform environments. In a rather explicit passage, Clark emphasizes such a constructivist side of his approach when he says that “[o]ur brains make the world smart so that we can be dumb in peace” (Clark, 1997, p. 180). One example he discusses in this regard is that of markets, which Clark sees as constructed environments that “scaffold” agents' cognition and foster their economic rationality. Following this line of argument, Clark's environmental interventionism has been explicitly related to niche constructionism by Sterelny (2004) and to autopoiesis by Di Paolo (2009). In this latter view, agents are considered engaging in a constructive, dynamic relationship with the environment in a way that makes life itself self-sustaining. Rather than with better known bounded and ecological rationality, the constructionism of extended cognition may more easily dovetail with what Shira Elqayam has called “grounded rationality” (Elqayam, 2011)^6^. In grounded rationality, environments acquire their normative status—i.e., ultimately decide whether a cognitive process or behavior is rational or not—only after being constructed as epistemic niches.

## Embodied Rationality: The Radical Embodied Approach To Rationality

### Three Challenges for Embodied Rationality

To define radical embodiment, we are faced with the same conceptual difficulties encountered in defining other embodied views and, possibly, even more. A commentator has remarked that “what is common to all versions of radical embodiment is that an agent's possession of her bodily anatomy is taken to be a constitutive part of her mind, in violation of neurocentric assumptions” (Jacob, 2016, p. 44), a definition that as such would also fit what has been called biological embodiment. Although biological embodiment is certainly required for embodied radicalism, it is, however, not sufficient for it. More specifically, Chemero (2011) defines radical embodiment as “the thesis that cognition is to be described in terms of agent-environment dynamics, and not in terms of computation and representation” (p. x). Thus, if we are looking for the core of radical embodiment, anti-representationalism and anti-computationalism are the places to look. Defined this way, we can appreciate how diametrically opposed radical embodiment is to Simon's representational and computational view of cognition. In this section, we use the banner “embodied rationality” (Mastrogiorgio and Petracca, 2016; Gallagher, 2018) for exploring the idea of rationality informed by radical embodiment that, as such, results the most conceptually distant from Simon's.

In pursuing embodied rationality, we face at least three challenges not encountered before. The first, and arguably the main one, is that embodied rationality has no benchmark of rationality to refer to, or, stated otherwise, no extant idea of rationality to build upon, reform, complement, or refound. While Simon's and Gigerenzer's views have been taken so far as conceptual platforms to be provided with new embodied foundations, embodied rationality has nothing preexisting to embody. To find an extant notion virtually compatible with radical embodiment, we should look for a kind of non-computational and non-representational approach to rationality, one that, as Rolla (2019) says, does not equate rationality with reasoning. But, as he adds, we have none of this sort:

Even the more unorthodox view known as Ecological Rationality, proposed for instance by Todd and Gigerenzer […], holds that a theory of rationality should describe the heuristic reasonings used by real agents, where heuristics involve following certain environmental cues and ignoring excessive information—which is a matter of reasoning nonetheless (p. 2).

For this reason, embodied rationality bears the privilege and the burden of writing its own history. The carte blanche it is given includes, importantly, also the liberty not to follow the usual framework of naturalistic approaches to rationality, adaptationism (see Neemeh, 2021), and therefore to conceive an altogether new definition of what is rational.

The second challenge concerns the intrinsic plurality of the radical field. If it is true that any embodied approach is internally plural, radical embodied cognition is even more plural. Gallagher (2009) has traced the precursors of radical embodiment to American pragmatism, classical phenomenology, and Wittgenstein's philosophy of language, to which can be added, more recently, ecological psychology, situated robotics, dynamical systems theory, and phenomenology-inspired neuroscience. And the list could be easily enlarged. In brief, anti-representationalism and anti-computationalism are only the common denominators of an array of radical positions that can variously inform embodied rationality.

Gallagher's rich list points to the third challenge for embodied rationality. Simon was, among other things, also an economist (of Nobel fame), and much of the debate over bounded rationality has been held in economics. Much of Gigerenzer's fame is also due to economics, for the controversy with Kahneman over the psychological foundations of behavioral economics. And even Clark's extended functionalism crossed paths, although briefly, with economics (Clark, 1997). In striking contrast, drawing upon such varied disciplinary backgrounds and having no extant notion of rationality to refer to, embodied rationality seems disciplinary disconnected from economics. Again, the privilege and burden of freedom.

In what follows, we will explore the idea of embodied rationality focusing in particular on two aspects. First, we will see how anti-representationalism and anti-computationalism—taken singularly or together—may radically transform the understanding of rationality. Second, we will see how embodied rationality can propel itself into unchartered routes discussing the view of rationality from the first-person perspective.

### Rationality Without Representations and/or Computations

As Rolla (2019) has put it, the challenge raised by radical embodiment to students of rationality is to figure out what “rationality without reasoning” means, which is tantamount to figuring out what a notion of rationality without mental representations and computations looks like. Stating the challenge this way may make one wonder whether representations and computations necessarily come as a bundle or might be thought of separately. In the latter case, radical versions of rationality based on computations but no representations, or, vice versa, on representations but no computations, could be envisaged. Indeed, computations and representations are usually understood as two sides of the same (cognitivist) coin, as it seems hard to think of representations that are not manipulated somehow or computations that have no content^7^. But following the incremental spirit of this article, we will attempt to disentangle their differential contribution to rationality.

Unlike Simon's approach in which representations and computations are equally central, in Kahneman's bounded rationality representations seem to feature more prominently than computations. In associative forms of judgment called System 1 (Kahneman, 2011), it is the content of the representation, and relatedly the semantic proximity of one representation to another, that guide the judgment. In addition, it is curious but telling that Kahneman's representationalism appeals to Freud's associationist concept of a symbol rather than to Simon's idea of symbols as objects of computation (Kahneman, 2011, p. 56; see Petracca, 2017). While associationist forms of reasoning are not bound to irrationality as Kahneman thinks, in so far as fast associations can be adaptive in the right context (see Gigerenzer, 2007), the point we wish to make is that they seem in any case to privilege the semantics of representations over the mechanics of computations.

Rodney Brooks has been one of the first to follow the alternative route, investigating how representations are not necessary for simple forms of intelligent behavior (e.g., Brooks, 1991). As a roboticist, he designed a class of goal-driven robots that, as has become customary to say, used “the world as their own best model.” This means that situated interactions with their proximal environments permitted Brooks' machines to accomplish their tasks without relying on representations—such as maps—of the environment. Brooks' robots (one of which was provocatively christened Herbert) were meant to be living falsifications of Newell and Simon's physical symbol system hypothesis, that is, the hypothesis that representations are necessary and sufficient conditions for intelligence (Newell and Simon, 1976). Of course, Brooks' robots were not free of computations, as Simon was eager to rebut (Vera and Simon, 1993), but they were not the kind of serial, centralized, vertically integrated, and content-based forms of computation that cognitivists advocated.

In Brooks' framework, the step from a non-representational form of intelligence to a non-representational form of rationality is not very long. Discussing Brooks' cognitive design, Susan Hurley explicitly speaks of rationality:

*Rationality* might emerge from a complex system of decentralized, higher-order relations of inhibition, facilitation, and coordination among different horizontal layers, each of which is dynamic and environmentally situated […] *Rationality* reconceived in horizontally modular terms is substantively related to the environment. It does not depend only on internal procedures that mediate between input and output, either for the organism as a whole or for a vertically bounded central cognitive module. Rather, it depends on complex relationships between dedicated, world-involving layers that monitor and respond to specific aspects of the natural and social environment and of the neural network, and register feedback from responses (emphasis added, quoted in Rolla, 2019, p. 4–5).

Yet, these remarks notwithstanding, it is not easy to single out non-representational forms of rationality in the biological domain that do not also qualify as forms of reasoning (remember, absence of reasoning is Rolla's requirement for radical embodied rationality). To have a sense of this difficulty, consider a heuristic discussed by Gigerenzer as a case of fast-and-frugal heuristic:

To measure the area of a candidate nest cavity, a narrow crack in a rock, an ant has no yardstick but a rule of thumb: Run around on an irregular path for a fixed period while laying down a pheromone trail, and then leave. Return, move around on a different irregular path, and estimate the size of the cavity by the frequency of encountering the old trail (Gigerenzer and Brighton, 2009, p. 107).

(If real ants did not already use such an embodied heuristic, it might very well have been devised by Brooks for his robot-ants). Now, the question is: does this heuristic involve any reasoning? While it likely does not involve representations, this is not enough for disqualifying it as a form of reasoning. In fact, commenting on this very example, Arnau et al. (2014) maintain that “these problem-solving activities qualify as instances of genuine reasoning” (p. 57). And Rolla (2019), as seen, seems to maintain that any use of heuristics qualifies ipso facto as reasoning. Now, if we agree that non-representational heuristics such as ants' qualify as (minimal) forms of reasoning, we should ask what we need more of (or, perhaps, less of) to achieve rationality without reasoning.

The answer to this question usually given by theorists of radical embodiment is one: extended dynamics. To qualify as a genuine instance of non-reasoning in the radical embodied sense, cognitive processes leading to rational outcomes need to be understood not only as non-representational but also as dynamically extended. This means, in a nutshell, that processes leading to rationality originate in the continuous *interaction* between agents and their environments. On this view, neither internal nor external resources alone would be enough to explain the emergence of a rational outcome, and interaction becomes the new explanatory cornerstone (e.g., Gallagher, 2017). It may seem paradoxical that Simon, the advocate of representations as requirements for intelligence, provided a good example of interactionist explanation that, intriguingly, concerns once again ants. Simon (1996a) discusses the case of the path made by an ant on the sand and wonders why the path is not regular:

[The ant] has a general sense of where home lies, but he cannot foresee all the obstacles between. He must adapt his course repeatedly to the difficulties he encounters and often detour uncrossable barriers. His horizons are very close, so that he deals with each obstacle as he comes to it; he probes for ways around or over it, without much thought for future obstacles. It is easy to trap him into deep detours. Viewed as a geometric figure, the ant's path is irregular, complex, hard to describe. But its complexity is really a complexity in the surface of the beach, not a complexity in the ant (p. 51).

Although Simon made this example to emphasize the sometime prominence of the environment (the beach, in this case) over agents' cognition in the explanation of complex behavior, his argument can be plausibly understood as if he meant that neither features of the ant nor those of the beach (for different sorts of insects could produce different trajectories) can explain alone the irregular path. Using the words of radical theorists, it can be said that the ant-beach pair forms a “coupled system.” Importantly, according to the radical embodied position, the ant-beach system does not merely explain the ant's path, but altogether forms an autonomous cognitive system that constitutes navigational abilities in that circumstance. Compared to the extended notion of constitution encountered in the discussion of the extended mind (section Extended Rationality: Extended Cognition and Un-Bounded Rationality), the idea of constitution held in radical embodiment is more specifically of the interactive, dynamic kind (see Gallagher, 2017).

### Rationality in the First-Person Perspective

If the hypothesis of rationality without reasoning seems outlandish enough, this section discusses the possibly more challenging hypothesis that rationality concerns the first-person rather than the third-person perspective. This issue was at the center of an epoch-making controversy in the 1970s between Simon, a staunch advocate of third-person-ism, and supporters of first-person-ism led by phenomenologist Hubert Dreyfus. Dreyfus' book *What Computers Cant' Do* (Dreyfus, 1972) has represented one of the most radical criticisms ever raised against Simon's thought, one that Simon's biographer says “left him angry, sad, and uncharacteristically silent” (Crowther-Heyck, 2005, p. 271). It took 20 years before Simon felt compelled to reply to Dreyfus' sort of criticism (Vera and Simon, 1993), when in the 1990s phenomenology was becoming one of the pillars of embodied cognition (see Petracca, 2017) One of the main points raised by phenomenologists concerned the impossibility to assess rationality objectively, “from the outside,” or, equivalently, from a third-person point of view. Famously stating that “intelligence must be situated” (Dreyfus, 1972, p. 62), Dreyfus introduced the idea of a “situation” as a construct critical and alternative to that of “context.” While contexts are objectively identifiable states of the world, situations are the outcome of a process of sense-making that can only be carried out by individuals. As Hans-Georg Gadamer put it,

[t]o acquire an awareness of a situation is, however, always a task of particular difficulty. The very idea of a situation means that we are not standing outside it and hence are unable to have any objective knowledge of it. We are always within the situation and to throw light on it is a task that is never entirely completed (quoted in Winograd and Flores, 1986, p. 29).

What does this mean for rationality? Using the words of another phenomenologist, Maurice Merleau-Ponty, situatedness means that “the world and reason are not problematic […] they are mysterious” (Merleau-Ponty, 2002, p. xxiii). Saying so, Merleau-Ponty appeals to the concept of “mysteriousness” as a way to counter the usual understanding of rationality popularized by Simon in terms of “problematicness,”^8^ according to which rationality is equivalent to the capacity of identifying unambiguous procedures to solve as much unambiguously identified problems (see Newell and Simon, 1972). Mysteriousness would instead emphasize the interactive, tentative, and above all non-pre-specifiable process of dealing with the world. On this view, conferring a behavior or an outcome the rationality status cannot be done on a third-person basis but becomes an eminently inter-subjective process, a “we” process. As Merleau-Ponty claims,

rationality is precisely proportioned to the experiences in which it is disclosed. To say that there exists rationality is to say that perspectives blend, perceptions confirm each other, a meaning emerges. But it should not be set in a realm apart, transposed into absolute Spirit, or into a world in a realist sense (Merleau-Ponty, 2002, p. xxii).

Gallagher (2018) has recently reintroduced the distinction between mystery and problem in the study of rationality, emphasizing that rationality “[is] not an observational or spectatorial stepping back that detaches from the situation to frame the world in abstract concepts” (Gallagher, 2018, p. 91). An important point Gallagher makes in this regard is that concepts such as problem-solving, reasoning, etc. should not be banished from the vocabulary of rationality, but rather reconceived:

the alternative [to the classical view of cognition and reasoning] is to think of mental skills such as reflection, problem solving, decision making, and so on, as enactive, non-representational forms of embodied coping that emerge from a pre-predicative perceptual ordering of differentiations and similarities (Gallagher, 2018, p. 86–87).

Here we meet again dynamics as a fundamental ingredient of radicalism (Chemero, 2011; Gallagher, 2017). If problem-solving is, as radical theorists insist, non-representational and non-pre-predicative—that is, if the range of solutions to problems cannot be predicated (let alone predicted) before engaging with the situation—interaction represents the only way for agents to cope with the complexity of the world and give proof of their skills.

As an example of embodied rationality, Gallagher discusses the rationality intrinsic in the use of the hand. Having encountered hands before in our discussion, we can see how now the tone is quite different. The hand seems to show a rationality of its own:

Consider, that there is a rationality that is implicit in the hand. […] As an agent reaches to grasp something, the hand automatically (and without the agent's conscious awareness) shapes itself into just the right posture to form the most appropriate grip for that object and for the agent's purpose. […] It is sometimes the case that very smart hand-brain dynamics take the lead over a more conceptual, ideational intelligence. For example, a patient with visual agnosia who is unable to recognize objects, when shown a picture of a clarinet, calls it a “pencil.” At the same time, however, his fingers began to play an imaginary clarinet (Gallagher, 2018, p. 88).

As another, quite different instance of embodied rationality, Gallagher et al. (2019) show that the dynamic perspective can be employed to explain the emergence of institutional forms of coordination such as markets, thus giving a radical twist to Clark's example of markets as extended forms of rationality (Clark, 1997). The variety of these examples just hints at the wide empirical applicability of the dynamic viewpoint.

It is of utmost importance to remark that insisting on dynamics and the first-person perspective does not take the inquiry of rationality out of the naturalistic heaven in which Simon placed it. In so far as phenomenology and other radical embodiment approaches are not only compatible but also an active part in the construction of a newly naturalized cognitive science (Gallagher and Varela, 2003), the same new naturalistic outlook can be transferred, we argue, into the naturalistic study of rationality.

## Discussion and Conclusion

This article has explored how increasingly radical views of embodied cognition may reform or even transform the idea of bounded rationality. To this purpose, four new embodied notions of rationality have been proposed (in increasing order of radicalism): embodied bounded rationality, body rationality, extended rationality, and embodied rationality. Although at this exploratory stage it would be too ambitious to provide self-contained definitions of these notions, their main features are displayed and juxtaposed in Table 1. In particular, they are arranged according to four criteria: degree of embodied radicalism, adherence to adaptationism as normative framework, reliance on mental representations, and view of heuristics. This juxtaposition allows us to propose some comparative remarks. The first remark concerns the extent to which the different notions of rationality question adaptation as a normative principle. While embodied bounded rationality (except in the case of embodiment of Kahneman's approach) and body rationality fundamentally retain Simon's adaptationist framework^9^, extended rationality seems more compatible with normative constructivism, which considers agents playing an active role in establishing normative standards through environmental manipulations. As for embodied rationality, it more resolutely goes down the post-adaptationist path (see Neemeh, 2021), although adaptation still seems to play a central role in Rolla (2019). For what concerns representations (and computations), embodied bounded rationality proposes to retain them by reforming their abstract nature, while body rationality and extended rationality rely on attenuated or intermittent (i.e., depending on the cognitive task) forms of representationalism. Downright rejection of representationalism is, instead, the trademark of embodied rationality. In this context, it is worth mentioning that a computational view based on the so-called “free energy principle” has recently tried to reconcile representationalism and anti-representationalism (see Constant et al., 2021), although it is doubtful whether it can be the last word on such a controversial matter. Finally, another comparative criterion concerns heuristics. Coherently with all the threads of bounded rationality (Kahneman's included), embodied bounded rationality and body rationality have heuristics as their main objects of inquiry, the only difference between them being that the former focuses on cognitive and neural heuristics, while the latter on body-based heuristics. Extended and embodied forms of rationality, in contrast, do not put special emphasis on heuristics as sources of rationality.

**Table 1:** Main features of the embodied approaches to rationality.

	**Degree of embodiment**	**Normative adaptationism**	**Representations**	**Heuristics**
Embodied bounded rationality	Moderate	Yes*^*a*^*	Yes	Embodied cognitive heuristics
Body rationality	Intermediate	Yes	Weak	Body-based heuristics
Extended rationality	Intermediate	Yes, but also normative constructivism	Yes, but not always necessary	Not the main source of rationality
Embodied rationality	Radical	No	No	Not the main source of rationality

a *Except in the case Kahneman's approach is embodied*.

Another point deserves discussion: what kind of evidence can be brought in support of one or the other form of rationality? It seems reasonable, at this early stage, to consider one form of rationality more well-supported than the other depending on the empirical success of the underlying embodied approach. If this criterion is adopted, embodied bounded rationality and body rationality seem to be currently in a better position. But the study of rationality opens, we argue, an entirely new terrain on which the embodied approaches can compete. More radical embodied approaches can prove their merit in this new field in particular the more we switch from the explanation of phenomena at the individual level to those at the collective and social level. Just to make one instance, the study of rationality in institutional settings like markets seems to be addressable in new ways through radical embodied approaches (see Clark, 1997; Gallagher et al., 2019; Petracca and Gallagher, 2020).

Some words should finally be spent on one giant in this article: Herbert Simon. Katsikopoulos and Lan (2011) have argued with reason that one way or another all scholars interested in the naturalistic study of rationality “labor under Herbert Simon's spell” (p. 728). Our take in this article is that, however, when it comes to embodiment, Simon's spell may be a bit less enchanting. This is why rather than taking Simon as a source of inspiration for all the embodied approaches to rationality, we have emphasized his suitability as a reference base for measuring the embodied content of different rationality proposals. The most fruitful way for embodied approaches to stand upon Simon's shoulders is, we argue, dialectical and interactive, taking such a giant of thought as a reference with whom to be in constant dialogue in the spirit of advancing the study of rationality.

## Data Availability Statement

The original contributions presented in the study are included in the article, further inquiries can be directed to the corresponding author.

## Author Contributions

The author confirms being the sole contributor of this work and has approved it for publication.

## Conflict of Interest

The author declares that the research was conducted in the absence of any commercial or financial relationships that could be construed as a potential conflict of interest. The handling editor declared a past co-authorship with the author of the manuscript.

## Publisher's Note

All claims expressed in this article are solely those of the authors and do not necessarily represent those of their affiliated organizations, or those of the publisher, the editors and the reviewers. Any product that may be evaluated in this article, or claim that may be made by its manufacturer, is not guaranteed or endorsed by the publisher.

## Footnotes

^1^The adjectives “weak” and “strong” are often used to refer to the two poles of embodied cognition in place of, respectively, moderate and radical (see, e.g., Tirado et al., 2018; Khatin-Zadeh et al., 2021).

^2^For the idea that the body proper constitutes cognition and the difference between constitution and causality, see Shapiro (2019). The misattribution of the constitutive status to causal determinants of cognition is called “causal-constitution fallacy” (see Adams and Aizawa, 2010).

^3^Witness to the half-wayness of biological embodiment is it having common features with “physical,” “organismoid,” and “organismic” embodiment as defined by Ziemke (2003) but not being reducible to them.

^4^When numbers are understood as signifiers of numerousness, they are called numerals. It is plausible that numerals rather than numerousness itself trigger the behavioral responses associated with prominent numbers (see Mastrogiorgio and Petracca, 2014).

^5^Instances of Simon's “extended” approach can be found in his study of organizations, where he said that “organizations can expand human rationality” (Simon, 1996b, p. 72).

^6^Hatchuel (2001) sees constructionism as a possible route, among others, to expand what he calls “the unfinished program of Herbert Simon.”

^7^Miłkowski (2013) has recently substituted Fodor's famous “no computation without representation” with his own “no representation without computation.” Although the two mottos reach the same conclusion, they express different nuances.

^8^The distinction between “mystery” and “problem” was first made by philosopher Gabriel Marcel (see Gallagher, 2018).

^9^It should be remarked that adaptation is not always supported by adaptationist mechanisms. In embodied simulation, for instance, the way extant neural resources are reused for different functions is a case of “exaptation.” Nonetheless, the extent to which the various mechanisms lead to environmental fitness remains the ultimate normative criterion.
